# Glass FRP-Reinforced Geopolymer Based Columns Comprising Hybrid Fibres: Testing and FEA Modelling

**DOI:** 10.3390/polym14020324

**Published:** 2022-01-13

**Authors:** Ahmad Rashedi, Riadh Marzouki, Ali Raza, Khawar Ali, Niyi Gideon Olaiya, Mayandi Kalimuthu

**Affiliations:** 1 College of Engineering, IT & Environment, Charles Darwin University, Ellengowan Drive, Casuarina, NT 0810, Australia; 2 Department of Chemistry, College of Science, King Khalid University, Abha 61413, Saudi Arabia; rmarzouki@kku.edu.sa; 3 Department of Chemistry, Faculty of Sciences of Sfax, University of Sfax, Sfax 3029, Tunisia; 4 Department of Civil Engineering, University of Engineering and Technology, Taxila 47050, Pakistan; ali.raza@uettaxila.edu.pk (A.R.); khawarali187@gmail.com (K.A.); 5 Department of Industrial and Production Engineering, Federal University of Technology, Akure PMB 704, Nigeria; ngolaiya@futa.edu.ng; 6 Department of Mechanical Engineering, Kalasalingam Academy of Research and Education, Krishnankovil, Srivilliputhur, Anand Nagar 626126, Tamil Nadu, India; k.mayandi@gmail.com

**Keywords:** circular columns, glass-FRP stirrups, finite element modelling, ductility, empirical model

## Abstract

This study seeks to evaluate the effectiveness of glass-FRP-reinforced geopolymer concrete columns integrating hybrid fibres (GFGC columns) and steel bar-reinforced geopolymer concrete columns incorporating hybrid fibres (SFGC columns) under eccentric and concentric loadings. Steel fibre (SF) and polypropylene fibres (PF) are two types of fibres that are mixed into hybrid fibre-reinforced geopolymer concrete (HFRGC). Eighteen circular concrete columns with a cross-section of 300 mm × 1200 mm were cast and examined under axial loading up to failure. Nine columns were cast with glass-FRP rebars, whereas the other nine were cast with steel rebars. Using ABAQUS, a nonlinear finite element model was established for the GFGC and SFGC columns. The HFRGC material was modelled using a simplified concrete damage plasticity model, whereas the glass-FRP material was simulated as a linear elastic material. It was observed that GFGC columns had up to 20% lower axial strength (AST) and up to 24% higher ductility indices than SFGC columns. The failure modes of both GFGC and SFGC columns were analogous. Both GFGC and SFGC columns revealed the same effect of eccentricity in the form of a decline in AST. A novel statistical model was suggested for predicting the AST of GFGC columns. The outcomes of the experiments, finite element simulations, and theoretical results show that the models can accurately determine the AST of GFGC columns.

## 1. Introduction

The processing of Portland cement releases carbon dioxide as a byproduct. The latest studies have used green concrete, called “geopolymer concrete”, to minimize the environmental impact of concrete structures [1,2]. Even though many studies have looked into the durability and mechanical properties of geopolymer, the integration of fibre-reinforced polymer (FRP) rebars in geopolymer columns have yet to be thoroughly researched. The critical parameters of reinforced concrete (RC) samples such as ductility and strength may be compromised due to the corrosion of steel and decreased serviceability of concrete structures. FRP rebars are potential substitutes for steel reinforcement. Glass-FRP rebars are gaining regulatory approval and credibility due to their high corrosion resistance, thermal conductivity, better tensile strength, and low density. FRP reinforcement is more effective in corrosive environments since it increases service life, reduces repair and overall construction costs [3,4]. Before failure, FRPs are anisotropic materials with linear elastic properties in compressive as well as flexural members [5,6,7,8,9]. FRPs along with steel rebars augmented the AST and ductility of the concrete columns as compared to plain concrete columns [10,11]. To overcome the brittleness of the concrete material, steel and polypropylene fibres were integrated into geopolymer concrete and glass-FRP rebars in this work.

Researchers in structural engineering have recently become interested in the excellent performance of FRP rebars in reinforced concrete construction. In comparison to steel-reinforced columns, glass-FRP-reinforced concrete columns show higher lateral deflection [12]. Glass-FRP stirrups usually fail due to bond-slip in the columns [13]. Due to their linear elastic nature, the interaction graphs of FRP-RC columns do not depict the balancing points until failure [14]. Furthermore, the procedures for determining the minimum reinforcement ratio of FRP-RC columns have been provided in the literature to avoid brittle-tension failure of FRP rebars on the tension side [15]. The structural response of glass-FRP and steel-reinforced concrete columns is the same except for AST. The AST of glass-FRP reinforced columns is 7% less compared to steel reinforced columns [16]. The applications of glass-FRP rebars in the compression members perform better with concrete than that in the tensile members, so using glass-FRP rebars as a compression reinforcement in concrete is preferable [17]. In circular columns, the exchange of steel reinforcements for an equivalent number of glass-FRP rebars reduces AST and bending moment while marginally increasing specimen ductility under several loading scenarios [18]. Columns reinforced with glass-FRP rebars must have enough lateral confinement to bear maximum loads equal to or greater than steel-reinforced columns [19]. The failure efficiency of full-scale glass-FRP-reinforced columns is different depending on lateral volumetric reinforcement. The test results show that large-scale glass-FRP-reinforced columns fail at 0.7% due to longitudinal bar buckling, and at 2.7%, volumetric lateral reinforcement fails due to core concrete crushing and lateral confinement rupture [20]. Different empirical methods for estimating the compressive strength of glass-FRP-reinforced columns have been developed by taking into account the contribution of glass-FRP reinforcement [16,18,20]. The literature has shown that the moment and AST of glass-FRP-reinforced circular samples had low AST and moment as compared to steel-reinforced, otherwise identical samples [18]. The number and size of longitudinal glass-FRP rebars were augmented to improve the AST, axial strain, and resistant effectiveness of glass-FRP-reinforced hollow columns [21,22].

Hadi et al. [23] investigated the structural performance of geopolymer compressive samples confined by BFRP tubes and/or basalt-FRP rebars. According to the researchers, steel-reinforced geopolymer compressive samples had an AST that was 27% to 34% higher than that of steel-reinforced ordinary Portland cement (OPC) compressive samples, while their ductility was reduced by 16–27%. However, the geopolymer compressive samples reinforced and confined with basalt-FRP rebars and tubes augmented ductility by 4–7% while the AST was reduced by 5–19% compared to OPC columns confined with the same material. Danda et al. [24] investigated the effect of NaOH molarity using GGBS and found that increasing NaOH molarity enhanced axial strength (AST) and reduced axial deflection of compressive samples. The activation method used for the manufacturing of geopolymers results in the emission of greenhouse gases and more energy consumption, both of which offer a safety risk. Additionally, the curing temperature and duration have a considerable impact on geopolymer synthesis [25,26]. Saranya et al. [27] examined the dynamics of steel fibre-reinforced dolomite-GGBS geopolymer short compressive samples under compressive loading and discovered that they had greater AST than steel fibre-reinforced OPC compressive samples. Furthermore, dolomite-GGBS geopolymer compression samples without ductile detailing to provide appropriate ductility and toughness exhibited similar performance to OPC compression samples, resulting in a lower cost-to-strength ratio. Maranan et al. [28] examined the response of glass-FRP reinforced geopolymer compressive samples, indicating that these compressive samples exceeded their OPC counterparts in terms of compressive strength.

Due to reducing the time and costs of research, the most important methods for a technical aspect to characterize the interaction process, the diversity of the failure mechanism of FRPs, and the unique response between FRPs and concrete are finite element models (FEMs) [29]. Excessive deformations and local buckling regulate the structural integrity of glass-FRP-reinforced samples, according to tests and finite element simulations [30,31]. Numerical modelling can accurately describe the structural efficiency of glass-FRP columns under buckling load [32,33]. Finite element simulations were used to simulate the efficiency of glass-FRP reinforced short concrete columns with varied slenderness ratios [34,35]. Initial failure mode and post-buckling efficiency test results are very comparable to finite element analysis. The numerical models have a high accuracy in evaluating the load-deflection response and failure modes of glass-FRP-reinforced columns [4,36,37]. Even though some authors have attempted to compare the response of glass-FRP-reinforced hybrid fibre-reinforced geopolymer concrete (HFRGC) columns to plain concrete, none of them have looked at the efficiency of such elements integrating hybrid fibres. The primary goal of this study is to use investigations and nonlinear finite element simulations to investigate the structural response of GFGC columns under eccentric and concentric stresses.

The SFGC columns were compared to GFGC columns in terms of structural efficiency. Different reinforcing lateral tie spacing, hybrid fibres, loading eccentricity, and loading circumstances were all examined. FEM was given using a modified concrete damaged plasticity (CDP) model. A novel theoretical model for the assessment of the AST of GFGC columns was also proposed. When highly corrosive and light glass-FRP composite materials are utilized in concrete columns, the findings of this study may be valuable to the concrete industry.

## 2. Materials and Methods

### 2.1. Materials

#### 2.1.1. Fibre-Reinforced Geopolymer Concrete

**Figure 1:**
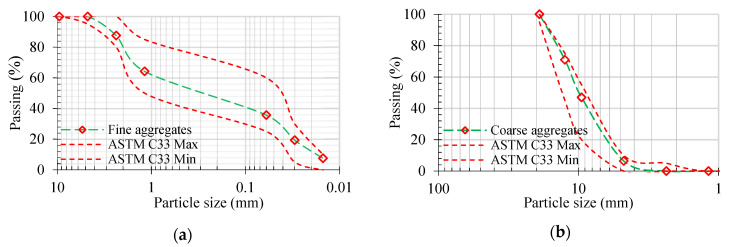
Sieve analysis of (**a**) fine aggregates (**b**) coarse aggregates.

Table 1 illustrates many characteristics of coarse aggregates. Coarse aggregates have a maximum size of 10 mm. Lawrencepur sand, which is abundantly available in the area, is used as fine aggregate. The fineness modulus and apparent particle density are 2.25 and 2586 kg/m^3^ respectively. The granular analysis of coarse aggregates and sand used in this research is illustrated in Figure 1. The diameters of SF and PF were 0.55 mm and 24 µm, and their lengths were 25 mm and 12 mm respectively. To enhance the geopolymer mix’s workability, a superplasticizer named Sika ViscoCrete^®^-3425 was used. Using a trial testing approach, the mix design of geopolymer (given in Table 2) with a nominal density of 2400 kg/m^3^ was obtained. In GFGC, two readily available waste materials were used as binders, i.e., 45% class F fly ash and 55% GGBS. As an activator, a mass ratio of 1:2.5 was used for a mixture of NaOH (14 M molarity) and Na_2_SiO_3_. The workability test performed according to ASTM C143 [38] reported a 125 mm slump of fresh GFGC. The setting time of the geopolymer was ninety minutes according to ASTM C807-13 [39]. Six concrete cylinders of cross-section 150 × 300 mm were cast. Compressive strength was 32 MPa of the same mix after 28 days.

#### 2.1.2. Steel and Glass-FRP Rebars

The longitudinal reinforcement and transverse stirrups for SFGC specimens were made from #3 (9.5 mm) and #4 (12.7 mm) steel rebars, respectively. These diameters are preferably selected in low-seismicity zones [40,41]. The same number of rebars was applied as a longitudinal and lateral reinforcement for the manufacturing of GFGC specimens as in SFGC. E-glass fibres coated with additives, fillers, and thermosetting vinyl ester resin were used to make the painted glass-FRP rebars. Ligatures were employed in this investigation, with SupAnchor^®^ fibres accounting for 80% of the volumetric quantity. The engineering properties of steel and glass-FRP rebars are summarized in Table 3.

### 2.2. Specimen Details

**Figure 2:**
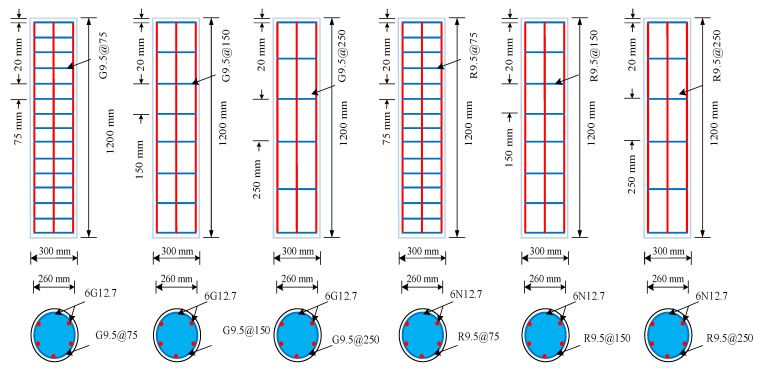
Geometrical parameters of manufactured specimens.

Eighteen (18) circular HFRGC columns were cast in this study. Nine of those were used for glass-FRP rebars and the other nine were for used steel rebars. Stirrups were used for lateral reinforcement for all 18 samples. The goal of the specimen testing was to see how the axial efficiency of columns was affected by hybrid fibres, reinforcing type, tie spacing, and loading eccentricity. The SFGC columns were cast as control specimens. All the columns had a cross-section of 300 mm × 1200 mm. These columns can fit into a testing machine but are sizable enough to be referred to as full-size samples. Figure 2 presents the details of the manufactured specimens. Six 12.7 mm-long rebars were used in each GFGC column, resulting in a reinforcement ratio of 1.10, which is ideal for non-seismic low-rise buildings. As a lateral reinforcement, #3 glass-FRP stirrups were used, positioned 75, 150, and 250 mm apart, resulting in volumetric ratios of 1.10%, 0.55%, and 0.32%, respectively. Correspondingly, six #4 long rebars were used to reinforce the SFGC columns, yielding a 1.10% reinforcement ratio. For transverse reinforcement, #3 steel stirrups were employed at a position of 75 mm, 150 mm, and 250 mm, giving volumetric ratios of 1.10%, 0.55%, and 0.32%, respectively. The concrete cover provided to all columns was 20 mm. Table 4 lists the geometric and testing details of the samples. The column framework was fabricated with PVC pipes with an inner diameter of 300 mm and a wall thickness of 5 mm. After the installation of the reinforcement cages, the HFRGC was poured into the mold and vibrated continuously with a poker. The samples were cured at room temperature.

### 2.3. Testing Arrangement

**Figure 3:**
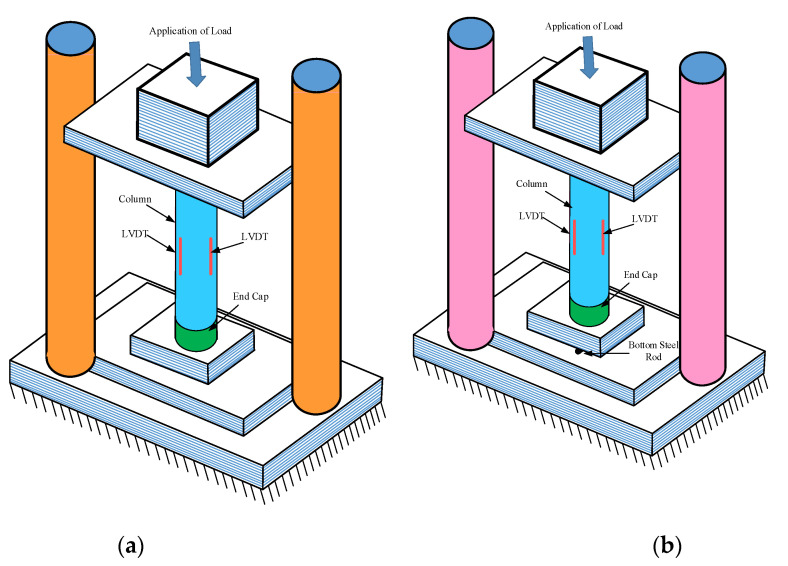
Experimental setup of specimens (**a**) concentric specimens (**b**) eccentric specimens.

The columns were analysed employing compression testing equipment with a load capacity of 5000 kN. All of the specimens were loaded at a rate of 0.003 mm/s. A plaster capping with a wall thickness of 10 mm and a width of 100 mm was provided at the top and bottom to prevent end crushing and non-uniform load distribution on the cross-section during sample testing. A consistent compressive force was delivered to the top surface of the concentrically loaded sample. In an eccentrically loaded sample, as shown in Figure 3, a load was applied at the appropriate eccentricity via a steel rod [41,42,43]. Three LVDTs were installed at 120° apart. The mechanical properties, such as longitudinal strain, longitudinal deflection, and compressive loads, were documented automatically by using a data logger of the testing machine.

## 3. Finite Element Modelling

**Figure 4:**
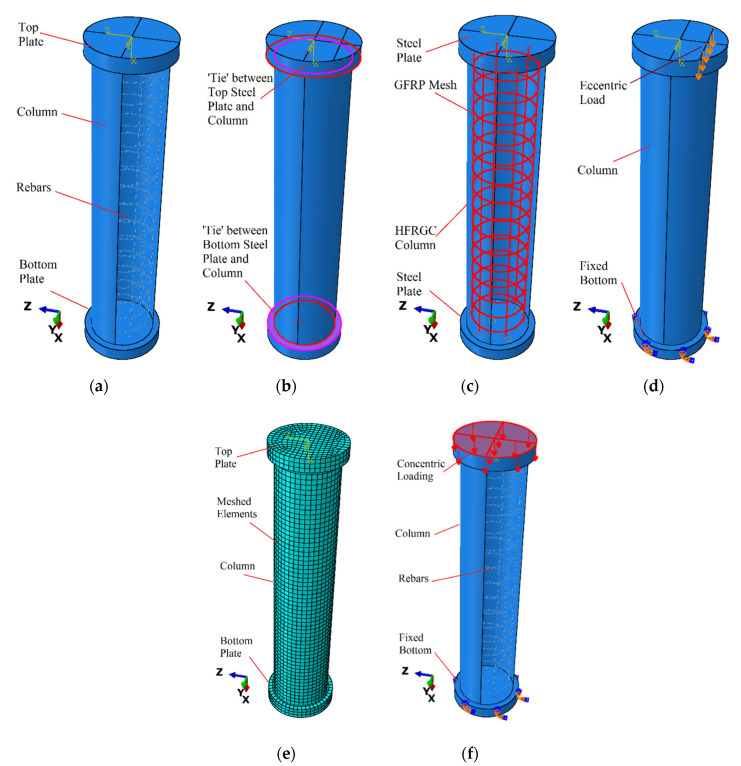
Simulated control model (**a**) geometry (**b**) interaction between steel plates and column (**c**) ‘embedded region’ between reinforcement and HFRGC (**d**) eccentric loading (**e**) meshed elements (**f**) concentric loading.

This section explains how to forecast the structural performance of GFGC and SFGC columns under axial loads using detailed finite element modelling of the complicated damaging efficiency of HFRGC and glass-FRP rebars. ABAQUS 6.14 was used to perform finite element simulations of samples. The columns’ ultimate failure mechanism, ultimate strength, initial stiffness, crack propagation efficiency, and post-failure stiffness efficiency are all taken into account in the proposed FEM. The FEM was standardized using the findings of a control specimen’s experiment (GFGC75-0). Three-dimensional solid stress sections were used to model the HFRGC. Three-dimensional deformable truss members were utilized to simulate the glass-FRP and steel reinforcing rebars. The complex efficiency of HFRGC was examined by using an improved concrete damaged plasticity model. The compression samples were restrained at the bottom end. The ‘embedded region’ constraint, which connects the compatible degrees of freedom (DOF) of the wire elements to the needed DOF of the 3D stress elements of concrete, was used to model the contact between the HFRGC and reinforcing material [4]. A uniformly distributed compressive force was supplied to the upper region of the concentrically loaded columns. The displacement control method was utilized to impose a line load of the same magnitude at the required eccentricity, validating the identical loading circumstances used in the experiments. The ‘tie’ constraint in ABAQUS was applied to fasten 50 mm-thick steel plates at the top and bottom surfaces of the samples for the application of boundary conditions. The boundary conditions that were applied to the simulated specimens were stated in Figure 4.

### 3.1. Modelling of HFRGC

To anticipate the structural efficiency of concrete elements, a precise definition of HFRGC in FEM is necessary. The elastic efficiency of HFRGC was described in this study using a Poisson’s ratio of 0.2 [44] and an elastic modulus of $4734\sqrt{f_{c}^{\prime }}$ [45]; where $f_{c}^{\prime }$ is the compressive strength of HFRGC, which is 32 MPa. By combining isotropic damaged elasticity, isotropic compressive plasticity, and isotropic tensile plasticity, the CDP model can accurately reflect the nonlinear and irreversible degradation of plain concrete. The CDP model is applied to model the complicated damaging efficiency of HFRGC in the current investigation, with minor adjustments as suggested by Chi et al. [46]. The use of the Chi et al. [46] model for numerical simulations of HFRGC is certified with equitable accuracy after careful review and evaluation between the calculations and experimental results.

The viscosity parameter (μ), yielding of the surface ($K_{c}^{hf}$), the biaxial stress to uniaxial stress under compression $\left(\sigma_{bo}^{hf}/\sigma_{co}^{hf}\right)$, the eccentricity (e), and the dilation angle of HFRGC (ψ) can all be defined in the CDP model to model the plasticity efficiency of HFRGC. As stated in the validation section of the presented work, the parameter was calibrated to ensure optimum results in comparison to the test data, and the other parameters in the CDP model for HFRGC were changed. For both plain and HFRGC, the default value for the parameter ‘e’ is always 0.1. The parameter $\sigma_{bo}^{hf}/\sigma_{co}^{hf}$ for HFRGC in the CDP model can be represented by Equation (1) [46].

(1) $$\frac{\sigma_{bo}^{hf}}{\sigma_{co}^{hf}}=\frac{k_{t}^{2}}{0.132k_{c}}\left[\left(0.728-\frac{0.749}{k_{t}}\right)+\sqrt{{\left(0.728-\frac{0.749}{k_{t}}\right)}^{2}+\frac{0.03}{k_{t}^{2}}}\right]$$

where $k_{c}$ and $k_{t}$ are the constants for compression and tension meridians, respectively. Because of the scarcity of experimental studies in this area, the constant *k_c_* has yet to be established for HFRGC. Therefore, the correlation for these constants can be represented by Equations (2) and (3), respectively [44].

(2) $$\;k_{c}=1+0.056\lambda_{sf}$$

(3) $$\;k_{t}=1+0.080\lambda_{sf}+0.132\lambda_{pf}$$

In these expressions, $\lambda_{sf}$ and $\lambda_{pf}$ are the fibre reinforcement values for SF and PF, respectively which can be defined by the mentioned expressions.

(4) λsf=Vsf(lsfdsf)

(5)  λpf=Vpf(lpfdpf)

where $V_{sf}$ shows the volumetric fraction of SF, $V_{pf}$ shows the volumetric fraction of PF, $l_{sf}$ shows the length of SF, $l_{pf}$ shows the length of PF, $d_{sf}$ shows the diameter of SF, and $d_{pf}$ shows the diameter of PF. The ratio of length to the diameter of fibres is known as the aspect ratio. After incorporating all the values of several parameters in Equation (1), the obtained value of $\sigma_{bo}^{hf}/\sigma_{co}^{hf}$ that was considered in the present work was 1.48. It is should be noted that the value of $\sigma_{bo}/\sigma_{co}=1.16$ is obtained when $k_{c}=1$ and $k_{t}=1$ are used to demonstrate the plain concrete.

As a result, the constants’ relationships can be represented. The shape factor (*K_c_*) for plain concrete is typically in the range of 0.64 to 0.80 [47]. When plain concrete is subjected to low hydrostatic stresses, the strength experimental measurements are shown to be very close. Furthermore, the value of 0.70 provides a more acceptable correlation when the concrete is subjected to high hydrostatic stresses [46]. As a result, for HFRGC, the relationship for $K_{c}^{hf}$ can be written in terms of the compressive and tensile meridians’ enhancing constants [44,46].

(6) $$\;K_{c}^{hf}=K_{c}\left(\frac{k_{t}}{k_{c}}\right)$$

Because the fibres are more resistant to crack opening and spreading, the tension meridian will be enhanced more than the compression meridian, i.e., $\frac{k_{t}}{k_{c}}\geq 1$. Consequently, the inequality $K_{c}^{hf}\geq K_{c}$ will always be true for the HFRGC.

The ψ is an important parameter that controls the flow rule. The value drops in the case of HFRGC, resulting in less plastic volumetric strain in the concrete matrix that effectively confines it. An equation for the dilation angle of HFRGC ($\psi^{hf}$) can be expressed in the form of $\lambda_{sf}$ and $\lambda_{pf}$ [46].

(7) $$\;\psi^{hf}=\psi \left(1-0.861\lambda_{sf}-0.097\lambda_{pf}\right)$$

According to the elastoplasticity hypothesis, which depicts the nonlinearity and irreversible deformation of concrete, the overall strain of concrete (ε) may be separated into two categories: elastic strain ($\varepsilon^{el}$) and plastic strain ($\varepsilon^{pl}$).

(8) $$\varepsilon =\varepsilon^{el}+\varepsilon^{pl}$$

**Figure 5:**
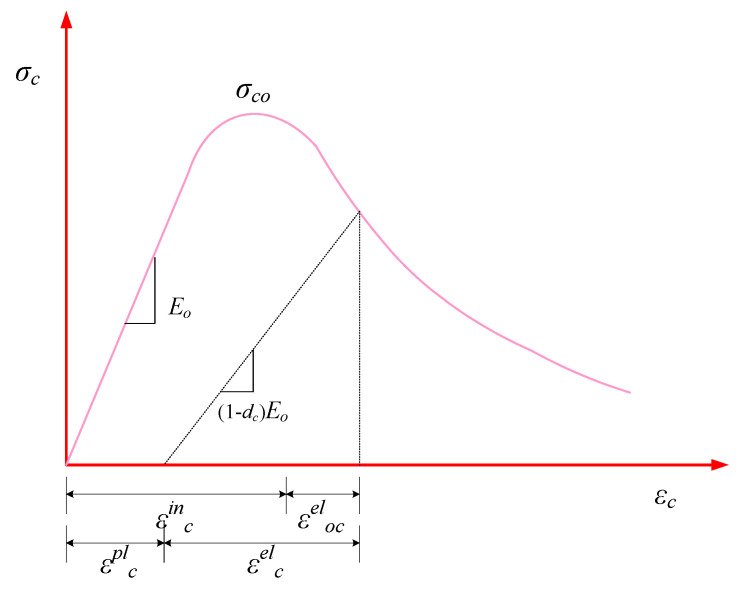
Stress-strain efficiency of HFRGC under compression.

Concrete nonlinearity can be attributable to either plasticity or damage, alone or a combination of both. Consideration of unloading stiffness is important for the simulation of the non-linearity of concrete. The deterioration of stiffness increases as strain increases, implying that the concrete damaging evolution is linked to the strain elements i.e., $\varepsilon^{el}$ and $\varepsilon^{pl}$ [46]. By involving two variables known as the uniaxial compression ($d_{c}$) and the uniaxial tension parameter ($d_{t}$), the CDP model in FEA defines the concrete damage. From Figure 5, the correlation for the compressive stress of plain concrete can be depicted by Equation (9).

(9) $$\sigma_{c}=\left(1-d_{c}\right)E_{o}\left(\varepsilon_{c}-\varepsilon_{c}^{pl}\right)$$

where $E_{o}$ is the elastic modulus of concrete defined according to ACI 318-95 [45], $\varepsilon_{c}$ is the concrete strain under compressive loading and $\varepsilon_{c}^{pl}$ is the plastic portion of the concrete strain under compressive loading. The parameter $d_{c}$ can be stated by Equation (10) as recommended by Wang and Chen [48].

(10)  dc=1e−1mc−1(e−εc, normin/mc−1)

where $m_{c}$ is the parameter that supervises the speed of compressive damage evolution having a value of 0.1 for the normal concrete [49], $\varepsilon_{c,\;norm}^{in}$ is the compressive inelastic concrete strain that could be expressed as: $\varepsilon_{c}^{in}/\varepsilon_{cu}^{in}$. Here, $\varepsilon_{cu}^{in}$ is the compressive strain corresponding to the inelastic concrete strain with a value of 0.033 [49]. Therefore, the modified compression-controlling parameter for HFRGC ($m_{c}^{hf}$) can be expressed by Equation (11) [46].

(11) $$m_{c}^{hf}=m_{c}\left(1+a_{m1}\lambda_{sf}+b_{m1}\lambda_{pf}\right)$$

where $a_{m1}$ and $b_{m1}$ are the constants linked to the characteristic parameter of fibres with magnitudes of 0.452 and 0.054, respectively [46]. Thus, for HFRGC, the compressive damaging parameter $\left(d_{c}^{hf}\right)$ and compressive stress ($\sigma_{c}^{hf}$) can be represented by Equations (12) and (13), respectively.

(12) dchf=1e−1mchf−1{e(−εc, normin/mchf)−1}

(13) σchf=[1−1e−1mchf−1(e−εc, normin/mchf−1)]Eo(εc−εcpl)

The ultimate compressive stress ($\sigma_{co}^{hf}$) and corresponding compressive strain ($\varepsilon_{co}^{hf}$) of HFRGC can be expressed by the following relations [46].

(14) $$\sigma_{co}^{hf}=\sigma_{co}\left(1+0.206\lambda_{sf}+0.388\lambda_{pf}\right)$$

(15) $$\varepsilon_{co}^{hf}=\varepsilon_{co}\left(1+0.705\lambda_{sf}+0.364\lambda_{pf}\right)$$

**Figure 6:**
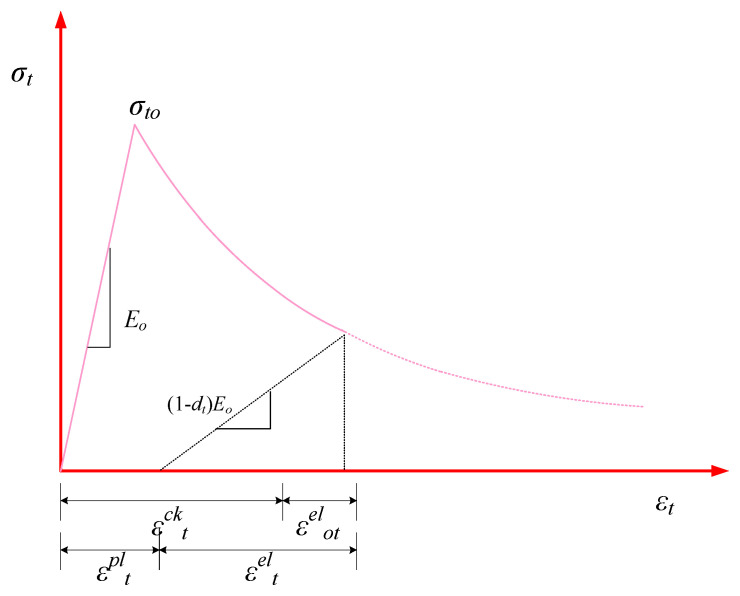
Stress-strain efficiency of HFRGC under tension.

A strain-hardening branch of the stress-strain curve characterizes the post-cracking efficiency of concrete. Strain-hardening represents the pre-ultimate efficiency of the stress-strain curve through plastic flow, while strain-softening represents the post-ultimate efficiency [50]. Figure 6 represents the tensile efficiency of concrete. The equation for tensile stress of concrete ($\sigma_{t}$) can be presented as:

(16) $$\sigma_{t}=\left(1-d_{t}\right)E_{o}\left(\varepsilon_{t}-\varepsilon_{t}^{pl}\right)$$

where $\varepsilon_{t}$ shows the concrete strain under tension and $\varepsilon_{t}^{pl}$ is the plastic region of the concrete strain under tension. The parameter $d_{t}$ can be represented by Equation (17) as proposed by Wang and Chen [48].

(17) dt=1e−1mt−1(e−εt, normck/mt−1)

where $m_{t}$ is the variable that controls the tensile damaging evolution speed, having a magnitude of 0.05 for plain concrete [49], and $\varepsilon_{t,\;norm}^{ck}$ is the normalized inelastic concrete strain under tension that might be expressed as: $\varepsilon_{t}^{ck}/\varepsilon_{tu}^{ck}$. Here, $\varepsilon_{tu}^{ck}$ is the strain corresponding to the normalized inelastic concrete strain under tension having a value of 0.1$\varepsilon_{cu}^{in}=0.0033$ [49]. As the speed of crack initiation and propagation is reduced in HFRGC, the modified tension controlling parameter for HFRGC ($m_{t}^{hf}$) can be expressed by Equation (18) [46].

(18) $$m_{t}^{hf}=m_{t}\left(1+a_{m2}\lambda_{sf}+b_{m2}\lambda_{pf}\right)$$

In this relationship, $a_{m2}$ and $b_{m2}$ are the variables linked to the characteristic parameter of PF and SF, with values of 0.156 and 0.628, respectively [46]. Thus, for HFRGC, the tension damage parameter $d_{t}^{hf}$ and tensile stress ($\sigma_{t}^{hf}$) can be expressed by Equations (19) and (20), respectively.

(19) dthf=1e−1mthf−1 {e(−εt, normck/mthf)−1}

(20) σthf=[1−1e−1mthf−1 (e−εt, normck/mthf−1)]Eo(εt−εtpl)

The ultimate tensile stress ($\sigma_{to}^{hf}$) and corresponding tensile strain ($\varepsilon_{to}^{hf}$) of HFRGC can be expressed by the following relations [46].

(21) $$\sigma_{to}^{hf}=\sigma_{to}\left(1+0.379\lambda_{sf}+0.020\lambda_{pf}\right)$$

(22) $$\varepsilon_{to}^{hf}=\varepsilon_{to}\left(1+0.498\lambda_{sf}+0.697\lambda_{pf}\right)$$

By considering the above relationship, the yield stress and the inelastic strain of HFRGC can be calculated for introduction into the FEA models.

### 3.2. Modelling of Glass-FRP and Steel Rebars

**Figure 7:**
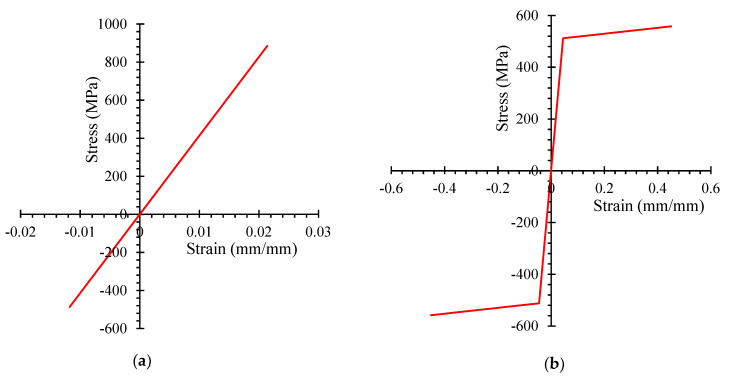
Reinforcement behaviors (**a**) linear elastic efficiency of glass-FRP rebars (**b**) elastic-plastic efficiency of steel rebars.

The glass-FRP and steel rebars were simulated as 3D truss elements. Table 3 shows the elastic moduli, yield strengths, and cross-sectional details of the reinforcements. For steel reinforcement, the Poisson’s ratio was 0.3 [5,51], while for glass-FRP reinforcement it was 0.25 [41]. The response of glass-FRP rebars was simulated up to failure by treating them as a linear elastic material [52,53,54]. Steel rebars were treated as an elastic-plastic material with comparable compression and tension properties to simulate their efficiency [51,55] as expressed in Figure 7. Compressive stress was found to be 55% of tensile strength in glass-FRP rebars [56].

### 3.3. Calibration of FEA Model

**Figure 8:**
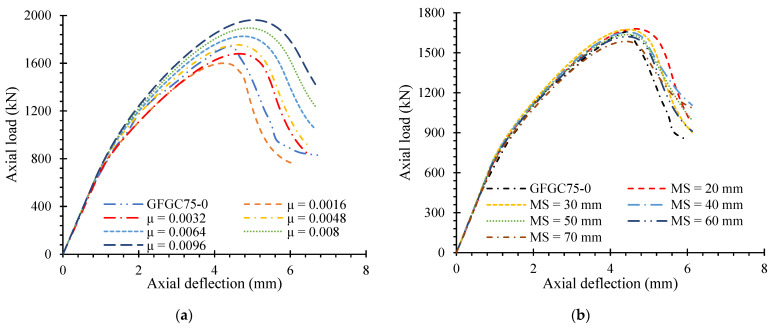
Effect of calibration on the behavior of the control sample (**a**) viscosity parameter (**b**) mesh size.

The experimental results were used to check the given FEM of the referenced specimen (GFGC75-0). The proposed FEM was scaled for different end conditions, HFRGC’s viscosity parameter (*μ*), multiple element types, and element mesh sizes to generate finite element assumptions that were close to the experiments. A similar relationship with the load-deflection efficiency of the reference sample was seen when all the DOF at the lower end of the samples was controlled while the upper end was able to translate and rotate on any axis. [57]. The effect of the parameter on the load-deflection response was observed to be remarkable, as shown in Figure 8a. The value of 0.0048 for μ demonstrated close agreement with the test results.

**Figure 9:**
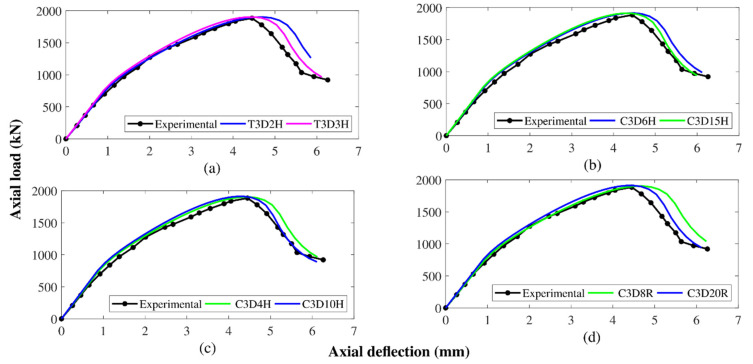
Results of calibration of different types of elements on the behavior of the control sample (**a**) truss types (**b**) triangular types (**c**) tetrahedral types (**d**) hexahedral types.

The mesh size was modified to provide the most optimum finite element calculations in a suitably limited time of FEA to minimize disruption in the circulation of strains and stresses in the HFRGC and glass-FRP rebars. The sensitivity of the load-deflection curve as a function of mesh size was shown in Figure 8b. Mesh sizes from 20 to 70 mm were investigated in this study. A similar agreement between the FEM model and experimental results was noted for a 40 mm screen size. The load-deflection response was used to investigate the effect of several kinds of the element of HFRGC and glass-FRP rebars in the control model. The studied kinds of the element of HFRGC were C3D8R and C3D20R for hexahedral elements, C3D4H and C3D10H for tetrahedral elements, and C3D6H and C3D15H for triangular elements, with C3D8R showing the highest accuracy as reported in previous works [4,37,46]. The glass-FRP and steel rebars were examined for T3D3H and T3D2H elements. A close correlation for the test results was observed when T3D2H elements were used for the reinforcing rebars. The effect of several element types is demonstrated in Figure 9.

## 4. Results and Discussion

### 4.1. Failure Modes and Cracking Patterns

**Figure 10:**
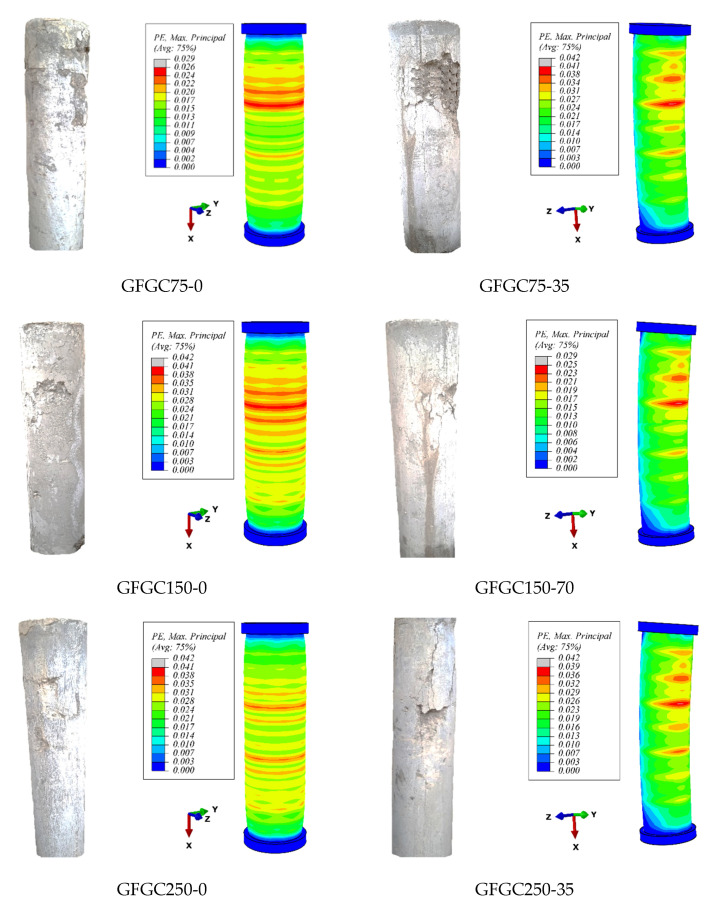

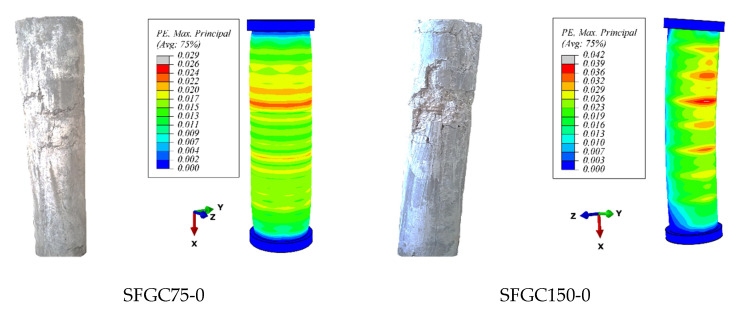
Overview of some GFGC and SFGC specimens after failure.

All the columns were noted to fail in the upper half. Figure 10 portrays the failure modes of some specimens after the test. Both groups of samples, i.e., GFGC and SFGC specimens, show similar failure modes and processes. Fracture in the longitudinal reinforcement is the major reason for GFGC column failure, while SFGC columns fail due to the buckling of long rebars. For the columns with a smaller distance between stirrups, the failure of the specimens was understood as the rupturing of stirrups and damage to the core. Conversely, the buckling of longitudinal rebars caused damage to the samples with a larger distance between stirrups. Up to 80% of ultimate load specimens behaved elastically after being loaded due to transverse confinement. The cracking of HFRGC started with a small vertical hairline fracture that was propagated from the upper corner of the columns after a further increase in load. Before reaching the ultimate load, cracks start propagating and their width expands. Cracks started to propagate along with the height of the specimen, and deflection augmented progressively after reaching the ultimate load. At this moment, the concrete cover was expanded, and the lateral stirrups were activated. When the AST of specimens decreased up to about 60% of the ultimate load, the lateral confinement reached the limit of its strength, triggering a rupture sound in the GFGC samples. The SFGC specimens triggered no rupturing sound of the steel rebars. The geopolymer concrete and hybrid fibres demonstrated a good bonding effect with the reinforcement rebars.

When the load is applied eccentrically on GFGC and SFGC samples, crushing of the concrete during failure starts from the compression side, while minor cracks appear on the tension side. Due to tensile forces acting normal to the plane of cracking and the bridging effect of fibres, concrete remains uncrushed from the tension side and no serious damage occurs in the concrete cover [58].

### 4.2. Ductility Index

**Figure 11:**
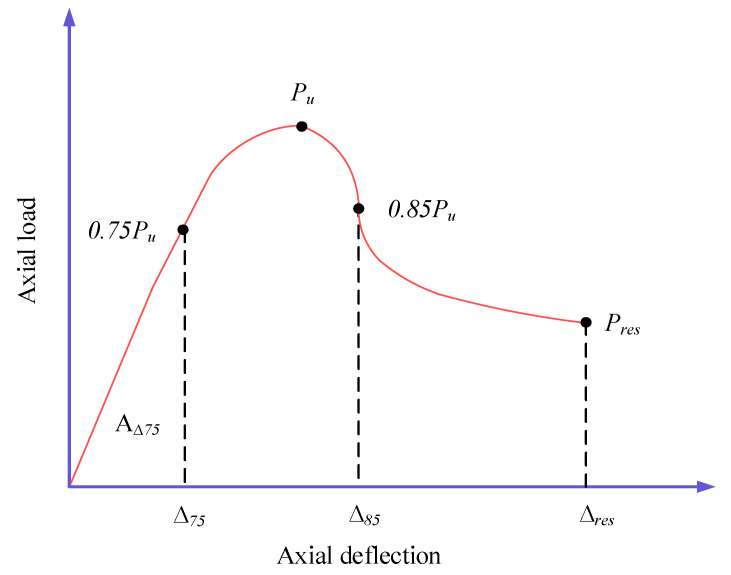
Ductility index measurement for specimens.

The rotation, axial deformation, curvature, strain, absorbed energy, and dissipated energy of elements are some of the variables that can be used to express ductility. The ductility value of the GFGC and SFGC samples was calculated utilising the following equation in this research [42,59,60]:

(23) $$I=\frac{A_{\Delta 85}}{A_{\Delta 75}}$$

where $\Delta_{75}$ is the value of the axial deflection of the specimen in the elastic section of the load-deflection curve corresponding to 75% of the peak load, and $A_{\Delta 75}$ is the area under the axial load-deflection curve up to 75%. As illustrated in Figure 11, $A_{\Delta 85}$ is the area under the axial load-deflection curve up to $\Delta_{85}$, where $\Delta_{85}$ is the value of the axial deflection of the columns in the post-ultimate collapse curve of the load-deflection corresponding to 85% of the peak load.

**Figure 12:**
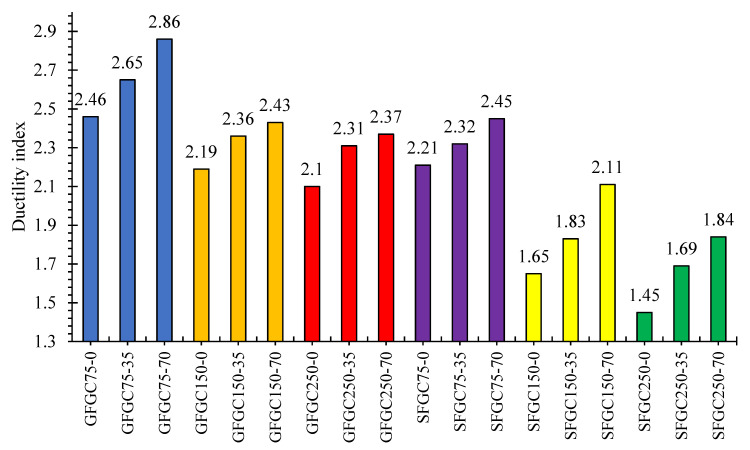
Ductility indices for several GFGC and SFGC specimens.

According to the literature, the GFGC specimens were found to be more ductile than identical SFGC specimens [42]. On average, the ductility values of GFGC columns were 24% greater than those of SFGC columns. The ductility values for all of the GFGC and SFGC columns that were evaluated are shown in Figure 12. The columns with a decreased stirrup distance had greater ductility indices, as expected. The average ductility indices were 2.66, 2.33, and 2.26 for the GFGC columns having a ligature distance of 75 mm, 150 mm, and 250 mm, respectively. Likewise, the ductility indices for the SFGC columns with a ligature distance of 75 mm, 150 mm, and 250 mm were 2.33, 1.87, and 1.66, respectively. The confinement of the concrete core is attributed to the reduced stirrup distance which restrained the long rebars and caused the improvement in ductility by absorbing more energy [60]. Additionally, the enhancement of the loading eccentricity of GFGC and SFGC columns is directly proportional to the ductility which can be observed from Figure 12.

### 4.3. Reinforcement Type Effect

The SFGC columns had 20% greater AST compared to the GFGC columns. The SFGC75-0 carried a peak load of 1949 kN at an axial deformation of 5.54 mm. The concentric SFGC column had an AST of 2240 kN with a center-to-center distance of 75 mm. In comparison to the concentric SFGC75 column, the identical GFGC75 column showed a decrease in AST, while its deflection was 14.9% compared to 6.6% in the SFGC75 column respectively. The AST of the concentrically loaded GFGC150 and GFGC250 exhibited values of 1651 kN and 1775 kN respectively, which were 74% and 87% of the AST of identical SFGC columns, respectively. The average axial deformation of the SFGC columns at the ultimate AST was 7% larger than that of the GFGC columns.

When compared to their GFGC counterparts, all of the eccentric SFGC columns had higher AST. The samples GFGC75-35 carry an AST of 1372 kN that was 23% lower than SFGC75-35. When loading eccentricity was augmented, GFGC columns had lower AST than SFGC identical columns. The AST of the GFGC specimen with a 150 mm stirrup distance was up to 54.5% lower while eccentricity was ameliorated from 35 mm to 70 mm. Identical SFGC columns reinforced with steel rebars showed a 39.3% reduction in AST when the eccentricity was augmented from 35 mm to 70 mm. As a result, the literature for plain concrete columns reveals that the SFGC columns surpass the GFGC columns in terms of AST [18,42].

### 4.4. Stirrup Spacing Effect

The literature shows that reductions in stirrup distance cause an increase in the AST of both SFGC and GFGC specimens [42,60]. The AST results for eccentric and concentric loading show good agreement with the literature. For concentrically loaded SFGC and GFGC columns, reducing the stirrup distance from 150 mm (by volume of 0.55%) to 75 mm (by volume of 1.10%) resulted in a percentage increase of 7.7% and 18% in AST, respectively. The confinement of the concrete core and longitudinal bar increases as the stirrup distance decreases, which causes the increment in AST of the specimens [60]. For SFGC columns, a percentage increase of 3.5% was seen when the stirrup distance was reduced from 250 mm (by volume of 0.32%) to 150 mm (by volume of 0.55%); however, the effect was the opposite for GFGC columns. AST and axial deflections were reduced when stirrup spaces were decreased for GFGC columns. In this research, AST was reduced by 6.9% when the distance was reduced from 250 mm to 150 mm for GFGC samples. Though the smaller stirrup distance is more preferable for glass-FRP-reinforced columns, this reduction in the AST may be ascribed to issues with testing the fabrication of the GFGC columns with a 75 mm stirrup distance.

### 4.5. Eccentricity Effect

**Figure 13:**
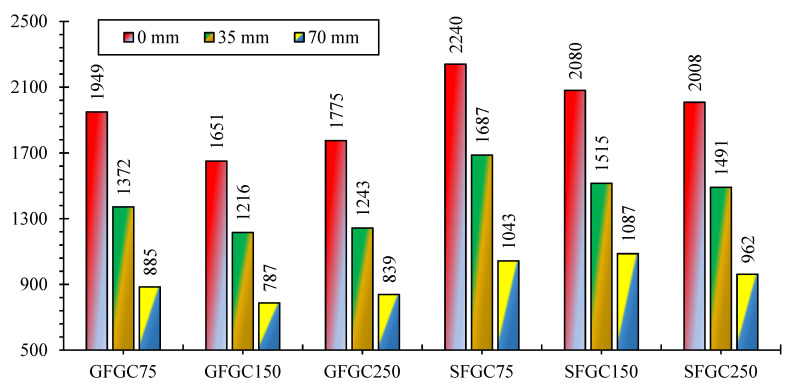
Influence of eccentric loading on the AST of samples.

The AST of all specimens was remarkably reduced as a result of the loading eccentricity. Figure 13 reflects the extent of eccentricity on the AST of SFGC and GFGC columns. When eccentricity was enriched from 0 mm to 35 mm and 70 mm, the AST of GFGC75 specimens decreased by 42% and 120%, respectively. When the eccentricity was adjusted by the same numbers, the AST of the specimens GFGC150 decreased by 35.7% and 109.7%, respectively. Similarly, raising the eccentricity from 0 to 35 and 70 mm reduced the AST of GFGC250 samples by 42.7% and 111.5%, respectively.

Similarly to GFGC samples, as the eccentricity of SFGC75 was increased from 0 to 35 mm and 70 mm, the reduction in AST was 32.7% and 114.7%, respectively. As in the GFGC150 samples, eccentricity augmented from 0 to 35 mm and 70 mm, the AST of SFGC150 was reduced by 37.3% and 91.3%, respectively. Furthermore, raising the eccentricity from 0 mm to 35 mm and 70 mm lowered the AST of the sample SFGC250 by 34.6% and 108.7%, respectively. Due to the loading eccentricity, both SFGC and GFGC specimens showed nearly the same amount of percentage losses in their AST.

## 5. Finite Element Analysis Predictions

### 5.1. Control Model

After confirmation for mesh size, element types, boundary conditions, and other HFRGC parameters, the reference FEM (GFGC75-0) showed variations of 2.6% and 6.8% for maximum AST and corresponding axial deflection from the tests. The elastic behavior, fracture patterns, and post-ultimate collapse efficiency of the reference sample were precisely described by the proposed FEM. Differences in the experimental and FEA testing environments, such as geometric imperfections, boundary conditions, material strength, sample manufacturing flaws, the differences in the features of glass-FRP rebars and steel rebars, as well as testing equipment accuracy, can be held responsible for minor discrepancies in the proposed FEM. The inconsistencies could potentially be attributed to FEA assumptions, such as assuming an ideal connection between HFRGC and rebars. The proposed FEM showed a strong connection with the tests and can be applied to analyse other SFGC and GFGC columns.

### 5.2. Ultimate Load against Corresponding Deflection

Table 5 summarizes the research observations and numerical indicators obtained using the proposed FEM for the GFGC and SFGC columns. The SFGC columns have larger ultimate loads than their GFGC counterparts in general. The largest discrepancy between test observations and FEM calculations was obtained for the ultimate AST of SFGC150-70 with a percentage error of 9.3%. The specimen GFGC250-70 had the largest variation in axial deflection at maximum AST with a percentage error of 24.5%. The specimens’ initial geometric flaws cause large variations in results that were not accounted for in their FEA models. Even though the FEM was confirmed using the experimental outcomes of a GFGC column (GFGC75-0), it anticipated the axial efficiency of SFGC specimens. The average percentage discrepancies between the experiments and FEA calculations for ultimate strength for GFGC and SFGC columns were 3.6% and 3.6%, respectively, while axial deflection discrepancies at ultimate strength were 7.8% and 6%, respectively. The majority of the FEM results underestimated the specimens’ ultimate AST.

The AST and deflection of eccentric columns were accurately identified using the recommended FEM. It captures the concentric SFGC column’s AST with a percentage inaccuracy of 4.5% and the eccentric GFGC column AST with a percentage inaccuracy of 4%. The tensile efficiency of HFRGC was exactly predicted in this research based on the accuracy of FEM predictions for eccentrically loaded SFGC and GFGC columns. Thus, the model of the tensile efficiency of HFRGC is needed for precise estimates of the eccentric columns. The difference between the experimental outcomes and FEM predictions for all HFRGC columns was 3.6% for ultimate AST and 6.9% for respective axial deflection. As a result of the safe under predictions for the AST of SFGC and GFGC columns, the suggested numerical model can be adopted and endorsed.

### 5.3. Load-Deflection Performance

**Figure 14:**
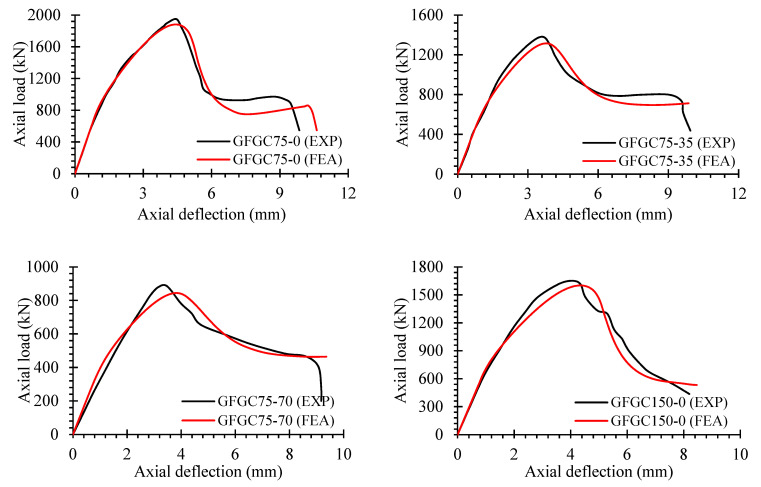

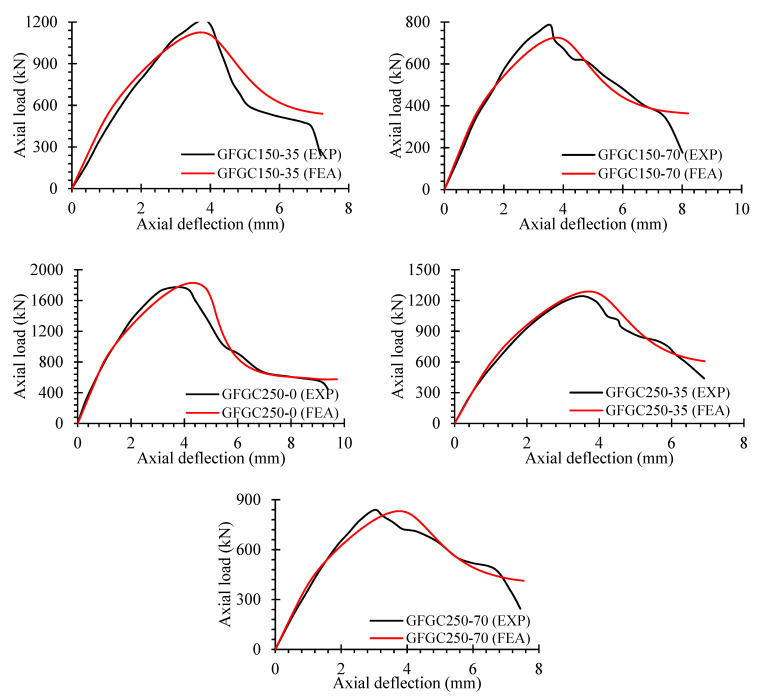
Comparison of experiments and FEA measurements for load-deflection curves of GFGC samples.

Figure 14 shows the analytical and simulation load-deflection curves of eccentrically and concentrically loaded GFGC samples. The efficiency of both eccentric and concentric GFGC columns was properly estimated by the recommended FEM in both the elastic and inelastic regions, with only slight deviations. FEM outcomes of load-deflection behavior for some samples, such as GFGC75-70 and GFGC150-35, are stiffer in the elastic range. The slight differences in the post-ultimate collapse efficiency of the samples between the experiments and FEM results could be related to the complicated damage and degradation efficiency of HFRGC after cracks have been started, and the interaction between glass-FRP rebars and HFRGC needs to be adjusted. Nevertheless, the entire load-deflection behavior seems to be realistically monitored by the recommended FEA models.

**Figure 15:**
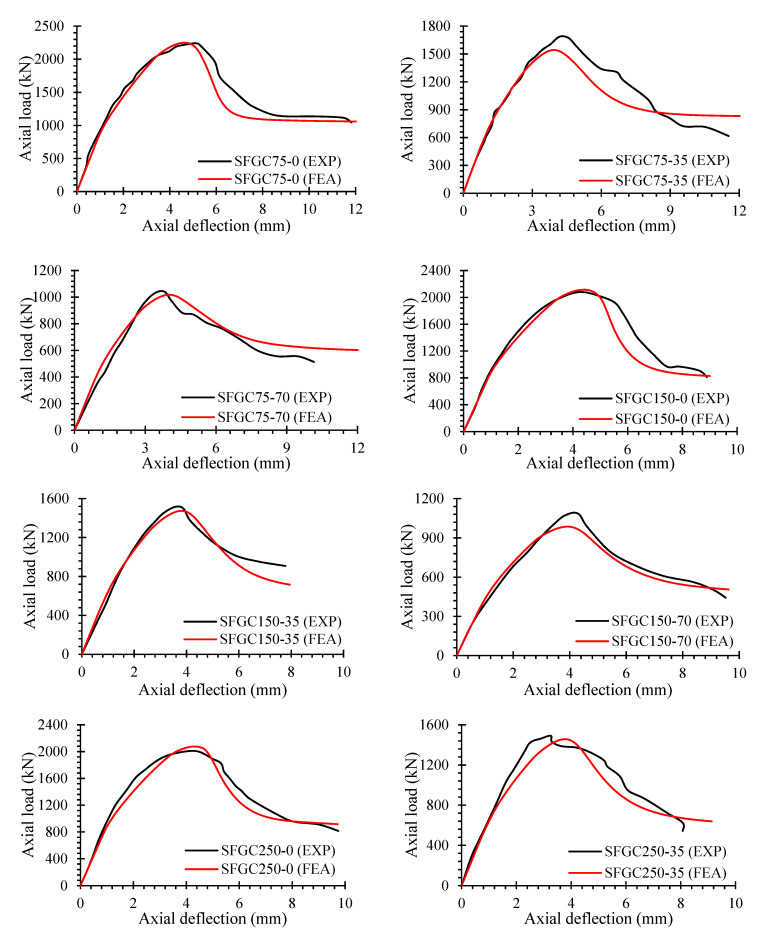

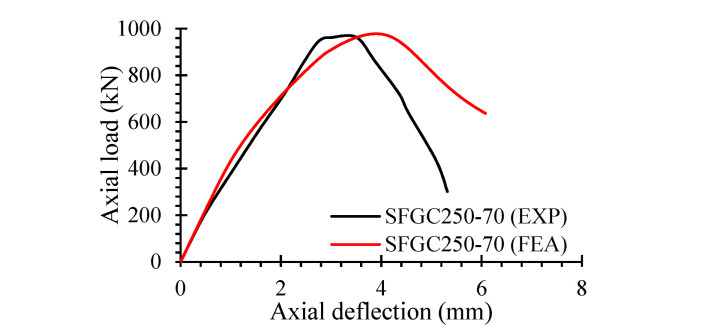
Comparison of experiments and FEA measurements for load-deflection curves of SFGC samples.

The load-deflection efficiency of all SFGC columns, as assessed by experimental studies and finite element simulations, is shown in Figure 15. The recommended FEM exactly predicted the load-deflection efficiency of all SFGC specimens in the elastic range. The samples SFGC75-35, SFGC150-35, and SFGC250-70 had some variations in the post-ultimate damage phase. Minor discrepancies in the proposed FEM can be attributed to differences in the experimental and FEA circumstances such as geometric imperfections, boundary conditions, material strength, specimen manufacturing faults, differences in the characteristics of glass-FRP and steel rebars, and testing equipment correctness. Because the proposed FEM could not successfully predict the post-ultimate efficiency of SFGC250-70, the specimen’s strength did not decline when the stirrups were opened.

### 5.4. Crack Patterns and Damage Behavior

The experimental failure and FEM crack patterns are shown in Figure 10. Cracks in HFRGC were displayed in the FEM using ultimate positive plastic strains. Since the orientation of the cracks is mostly perpendicular to these plastic strains, the cracking trends can be assessed effectively using these types of strains. Figure 10 shows that the recommended FEM satisfactorily captured the cracking efficiency of SFGC and GFGC specimens. The crack propagation and spalling of the concrete cover of the specimens began when the reinforcement began to yield after the ultimate load. As predicted by the measurements, the bulk of the specimens were damaged in the upper portion. It was also revealed that the recommended FEM accurately predicted the cracking patterns and failure modes of eccentric columns. The elastic modulus of glass-FRP rebars is lower than that of steel rebars, which is comparable to that of concrete. As a result, the glass-FRP rebars and HFRGC combination were more coordinated when the load was applied to GFGC specimens.

## 6. Theoretical Investigation

### 6.1. FRP-Confining Mechanism

**Figure 16:**
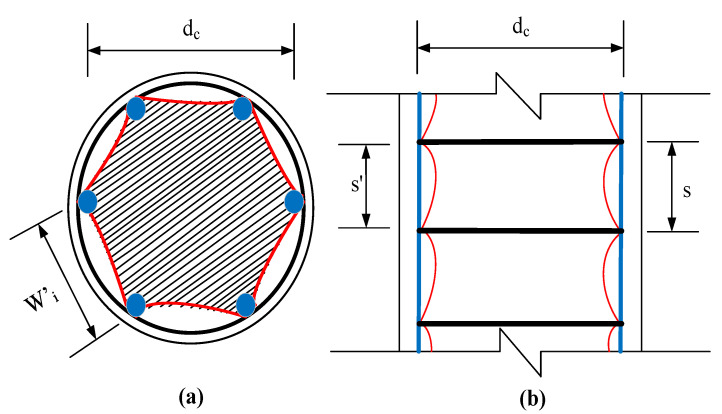
Effectiveness of glass-FRP confinement (**a**) cross-section (**b**) elevation of GFGC column.

Columns’ strength and ductility are enhanced by using glass-FRP stirrups to confine concrete [61]. The confinement effect is minor at the start, but it becomes active at the ultimate strength with increasing lateral strains that tend to result in lateral pressure. The lateral glass-FRP reinforcement prevents concrete dilatation caused by lateral pressure after an axial load is applied. The AST of glass-FRP-reinforced columns was previously computed without taking into account the resistance efficiency of glass-FRP stirrups. The predictions are underestimated since the influence of glass-FRP-confinement on the AST of columns is overlooked [6]. The lateral confining pressure ($f_{l}$) due to glass-FRP stirrups can be showed by Equation (24) [62].

(24) $$f_{l}=\frac{A_{ft}f_{fv}}{sd_{c}}$$

where $A_{ft}$ shows the area of glass-FRP stirrups, $d_{c}$ shows the diameter of the core, s shows the pitch glass-FRP stirrups, and $f_{fv}$ shows the stress in glass-FRP stirrups at $f_{co}^{\prime }$. This can be computed by employing the equation below [63]:

(25) $$f_{fv}=0.30{\left(\frac{E_{f}k_{e}\rho_{t}}{f_{co}^{\prime }}\right)}^{1.10}+26.6$$

where $E_{f}$ is the elastic modulus of glass-FRP stirrups, $\rho_{t}$ is the volumetric ratio of glass-FRP lateral stirrups, and $k_{e}$ is the confinement effectiveness constant. As illustrated in Figure 16, Mander et al. [62] suggested a correlation for this constant by incorporating a bending effect because of the confinement of transverse stirrups. The bending efficiency resembles the form of a second-degree parabola [61,62].

(26) $$k_{e}=\frac{\left(1-\sum_{i=1}^{n}\frac{{\left(w_{i}^{\prime }\right)}^{2}}{6d_{c}^{2}}\right){\left(1-\frac{s^{\prime }}{2d_{c}}\right)}^{2}}{1-\rho_{l}}$$

In this equation, $w_{i}^{\prime }$ is the clear distance between the longitudinal glass-FRP rebars, s′ is the distance between glass-FRP stirrups, and $\rho_{l}$ is the reinforcement ratio of long glass-FRP rebars in percentage. The functional transverse confining stress due to glass-FRP stirrups can be described by Equation (27) as recommended by Mander et al. [62].

(27) $$f_{le}=k_{e}f_{l}$$

The parameters $f_{l}$ and $k_{e}$ can be calculated using Equations (24) and (26), respectively. Ahmad et al. [64] and Raza et al. [65] recommended the correlations for calculating the peak stress and respective strain of concrete constrained with glass-FRP stirrups based on the empirical values given by Equations (28) and (29), respectively.

(28) $$\frac{f_{cc}^{\prime }}{f_{co}^{\prime }}=1+3.1{\left(\frac{f_{le}}{f_{co}^{\prime }}\right)}^{0.83}$$

(29) $$\frac{\varepsilon_{cc}^{\prime }}{\varepsilon_{co}^{\prime }}=1.85+7.46{\left(\rho_{k}\right)}^{0.71}{\left(\rho_{\varepsilon }\right)}^{1.17}$$

where $f_{cc}^{\prime }$ is the ultimate stress of concrete constrained with glass-FRP stirrups, $\varepsilon_{co}^{\prime }$ is the unconfined concrete compressive strain respective to ultimate compressive load and $\varepsilon_{cc}^{\prime }$ is the maximum concrete compressive strain confined with glass-FRP stirrups. The parameters $\rho_{k}$ and $\rho_{\varepsilon }$ are the confined concrete stiffness ratio and strain ratio which can be calculated as follows, respectively:

(30) $$\rho_{k}=\frac{\varepsilon_{h,rup}}{\varepsilon_{co}^{\prime }}$$

(31) $$\rho_{\varepsilon }=\frac{2E_{f}t}{(f_{co}^{\prime }/\varepsilon_{co}^{\prime })D}$$

### 6.2. Proposed AST Model

A series of simulations for measuring the compressive strength of FRP-RC columns has been recommended in previous research [16,20,66,67,68,69,70]. Most of these studies did not include the influence of glass-FRP resistant pressure, except for [66,67] which offered models for glass-FRP-reinforced conventional concrete specimens. The ultimate longitudinal strain of concrete is assumed to be equivalent to the compressive strain of glass-FRP rebars in a few of the strength models [66,68]. The experimental data reflects that the AST of GFGC columns was underestimated when the transverse pressure related to the lateral constraint of the concrete core was ignored. According to the comparative analysis, the current recommended model accurately depicts the AST of HFRGC columns. This is accomplished by calculating the restricting effect of glass-FRP stirrups and assuming that the ultimate stress and strain of HFRGC are the same as those of concrete constrained by glass-FRP. The ultimate AST of GFGC columns ($P_{n}$) was studied using a new experimental setup represented by Equation (32). This theoretical model is suggested based on the regression analysis and curve fitting tools in MATLAB based on the experimental results of the present study.

(32) $$P_{n}=0.85f_{co}^{\prime }\left(A_{g}-A_{FRP}\right)\left\{1+3.1{\left(\frac{f_{le}}{f_{co}^{\prime }}\right)}^{0.83}\right\}+\varepsilon_{co}^{\prime }E_{FRP}A_{FRP}\left\{1.85+7.46{\left(\rho_{k}\right)}^{0.71}{\left(\rho_{\varepsilon }\right)}^{1.17}\right\}$$

**Figure 17:**
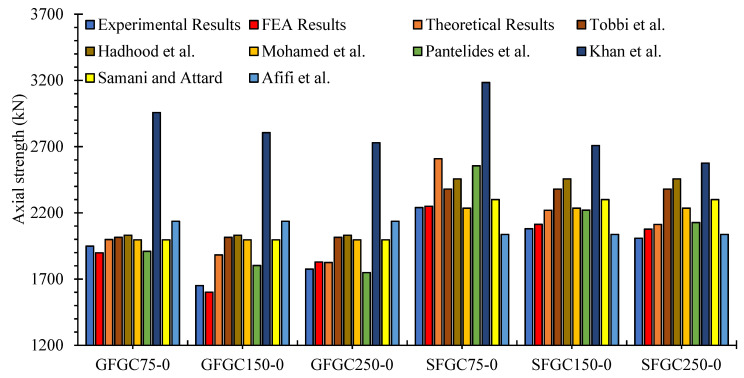
Comparison of testing results, theoretical estimates, and FEM predictions of ultimate AST of samples.

In this relation, $A_{g}$ is the whole area of the sample in the cross-section, $A_{FRP}$ is the overall area of glass-FRP long rebars in the cross-section, and $E_{FRP}$ is Young’s modulus of glass-FRP long rebars. For the SFGC and GFGC concentric specimens, the suggested model’s assumptions were equated to those of proposed models, experiments, and FEM predictions. Figure 17 represents the comparison of testing results, theoretical estimates, and FEM estimates for the ultimate AST of specimens. The theoretical models proposed by some previous studies [16,20,66,67,68,69,70] were used to assess and compare the accuracy of the predictions of the presently proposed models.

The current recommended model accurately reflected the AST of HFRGC-columns, according to the comparative analysis. The average differences of the estimates of the equations given by Mohamed et al. [20], Afifi et al. [16], Pantelides et al. [66], Khan et al. [67], Tobbi et al. [68], Samani and Attard [69], and Hadhood et al. [70] from the testing measurements were 11.6%, 9.9%, 16.4%, 88.5%, 21.8%, 16.9%, and 15.7%, respectively. The average difference between the several empirical model’s predictions and FEM’s predictions was 7.5%. The proposed FEA model and proposed theoretical model presented average errors of 0.6% and 8.1% from the experimental results of concentric SFGC and GFGC columns, respectively. Thus, the suggested FEA and empirical models may precisely predict the AST of GFGC columns by considering the contribution of glass-FRP lateral rebars and axial rebars.

## 7. Conclusions

In the present study, the experiments, FEA, and computational methods were used to investigate the structural efficiency of SFGC and GFGC columns. Following are some important observations from this research:

1. The GFGC columns had a lower AST than the SFGC columns, with an average difference of only 20%. This shows that the inclusion of hybrid fibres in geopolymer concrete can perform efficiently in glass-FRP-reinforced circular columns to obtain sustainable and environmentally friendly structural elements.
2. All of the GFGC and SFGC columns had the same failure mechanisms and proceeded through the same process. The collapse was mostly seen in the upper part of both eccentric and concentric columns. The GFGC columns were damaged due to a fracture in the longitudinal rebars, whereas the SFGC columns were damaged due to buckling of the main bars.
3. GFGC samples are more ductile than SFGC samples. GFGC samples had an average ductility value 24% higher than SFGC columns. The columns with a shorter stirrup distance exhibited greater ductility values due to well-confined long rebars and good entrapment of the concrete core to retain more energy.
4. The lessening in stirrup spacing improved the AST of both GFGC and SFGC columns. When the stirrup distance was reduced from 150 mm to 75 mm, the average percentage increase for both eccentric and concentric GFGC and SFGC columns was 15% and 6%, respectively. Reducing the stirrup distance from 250 mm to 150 mm resulted in a 5% increase in AST for SFGC columns, but a 5.5% drop in AST for GFGC columns.
5. The AST of both GFGC and SFGC columns was significantly reduced as a result of loading eccentricity. The AST of GFGC columns with 75 mm stirrup spacing was lowered by 42% and 120%, respectively, due to the application of loads at the eccentricity of 35 mm and 70 mm.
6. The proposed FEM predicted the efficiency of HFRGC with high accuracy by considering the hybrid fibre-dependent CDP model. The average errors represented by the proposed FEM for the AST and corresponding axial strain of studied specimens were 3.6% and 6.9%, respectively.
7. The proposed new theoretical equation for the AST of GFGC columns functioned well for the experimental measurements of examined samples by taking into account the axial participation of glass-FRP main rebars and the lateral confinement of glass-FRP stirrups. The recent research found that the average deviations of the predictions of the currently proposed theoretical model from experimental data and finite element analysis of AST of samples were 8.1% and 7.5%, respectively.

## Figures and Tables

**Table 1:** Several features of coarse aggregates.

Parameter	Value	Parameter	Value
10% fine value	132	Specific gravity	2.24
Apparent density	2589 kg/m^3^	Los Angeles abrasion	40.17%
Bulk density	1278 kg/m^3^	Maximum size	10 mm
Water absorption at 24 h	3.32%	Minimum size	4.75 mm

**Table 2:** Mix design of GFGC.

Material	Quantity (kg/m^3^)	Material	Quantity (kg/m^3^)
Coarse aggregates	1184	Fly ash	246
Sand	500	GGBS	162
Water	123	Superplasticizer	38
NaOH solution (14 M)	39	Na_2_SiO_3_	105
Steel fibres	23.62	Polypropylene fibres	1.92

**Table 3:** Engineering parameters of steel and glass-FRP rebars.

Parameter/Material	Diameter (mm)	Area (mm^2^)	Yielding Strength (MPa)	Young’s Modulus (GPa)	Ultimate Strain (%)
Steel rebars	9.5	71	428	210	4.85
	12.7	129	512	210	4.51
Glass-FRP rebars	9.5	70.8	822	50	2.24
	12.7	126.7	885	50	2.14

**Table 4:** Test matrix.

Specimen ID	Rebars Type	Longitudinal Rebars			Eccentricity (*e*) (mm)	Transverse Rebars			*e*/*D* Ratio
		Diameter (mm)	No. of Rebars	Reinforcing Ratio (%)		Diameter (mm)	Spacing (mm)	Vol. Ratio (%)	
GFGC75-0	Glass-FRP rebars	12.7	6	1.10	0	9.5	75	1.10	0
GFGC75-35					35				0.12
GFGC75-70					70				0.23
GFGC150-0	Glass-FRP rebars	12.7	6	1.10	0	9.5	150	0.55	0
GFGC150-35					35				0.12
GFGC150-70					70				0.23
GFGC250-0	Glass-FRP rebars	12.7	6	1.10	0	9.5	250	0.32	0
GFGC250-35					35				0.12
GFGC250-70					70				0.23
SFGC75-0	Steel rebars	12.7	6	1.10	0	9.5	75	1.10	0
SFGC75-35					35				0.12
SFGC75-70					70				0.23
SFGC150-0	Steel rebars	12.7	6	1.10	0	9.5	150	0.55	0
SFGC150-35					35				0.12
SFGC150-70					70				0.23
SFGC250-0	Steel rebars	12.7	6	1.10	0	9.5	250	0.32	0
SFGC250-35					35				0.12
SFGC250-70					70				0.23

**Table 5:** Testing and FEA results.

Sample ID	Experimental Results		FEA Results		% Age Difference in P_u_	% Age Difference in Deflection at P_u_
	Ultimate Load, P_u_ (kN)	Axial Deflection at P_u_ (mm)	P_u_ (kN)	Axial Deflection at P_u_ (mm)		
GFGC75-0	1949	4.54	1898	4.23	2.62	6.83
GFGC75-35	1372	3.51	1315	3.57	4.15	1.71
GFGC75-70	885	5.12	886	5.45	0.11	6.45
GFGC150-0	1651	3.91	1601	4.07	3.03	4.09
GFGC150-35	1216	3.61	1126	3.51	7.40	2.77
GFGC150-70	787	3.36	726	3.58	7.75	6.55
GFGC250-0	1775	3.55	1829	4.05	3.04	14.08
GFGC250-35	1243	3.32	1288	3.44	3.62	3.61
GFGC250-70	839	2.86	831	3.56	0.95	24.48
SFGC75-0	2240	4.84	2250	4.4	0.45	9.09
SFGC75-35	1687	3.94	1544	3.74	8.48	5.08
SFGC75-70	1043	3.53	1018	3.71	2.40	5.10
SFGC150-0	2080	4.09	2113	4.07	1.59	0.49
SFGC150-35	1515	3.37	1474	3.59	2.71	6.53
SFGC150-70	1087	4.03	986	3.7	9.29	8.19
SFGC250-0	2008	4.11	2077	4.13	3.44	0.49
SFGC250-35	1491	3.08	1454	3.45	2.48	12.01
SFGC250-70	962	5.15	978	5.51	1.66	6.99

## Data Availability

Some or all data will be available upon request from the corresponding author.
